# Experimental, molecular docking and molecular dynamic studies of natural products targeting overexpressed receptors in breast cancer

**DOI:** 10.1371/journal.pone.0267961

**Published:** 2022-05-10

**Authors:** Mohammad Sadegh Taghizadeh, Ali Niazi, Ali Moghadam, Alireza Afsharifar

**Affiliations:** 1 Institute of Biotechnology, Shiraz University, Shiraz, Iran; 2 Center of Plant Virology Research, Shiraz University, Shiraz, Iran; University of Akron, UNITED STATES

## Abstract

Natural compounds are proper tools for inhibiting cancer cell proliferation. Hence, the search for these ligands of overexpressed receptors in breast cancer has been a competitive challenge recently and opens new avenues for drug discovery. In this research, we have investigated molecular interactions between natural products and overexpressed receptors in breast cancer using molecular docking and dynamic simulation approaches followed by extraction of the best ligand from *Citrus limetta* and developing for nanoscale encapsulation composed of soy lecithin using a sonicator machine. The encapsulation process was confirmed by DLS and TEM analyses. Anticancer activity was also examined using MTT method. Among the investigated natural compounds, hesperidin was found to bind to specific targets with stronger binding energy. The molecular dynamics results indicated that the hesperidin-MCL-1 complex is very stable at 310.15 K for 200 ns. The RP-HPLC analysis revealed that the purity of extracted hesperidin was 98.8% with a yield of 1.72%. The results of DLS and TEM showed a strong interaction between hesperidin and lecithin with an entrapped efficiency of 92.02 ± 1.08%. Finally, the cytotoxicity effect of hesperidin was increased against the MDA-MB-231 cell line with an IC_50_ value of 62.93 μg/mL after encapsulation, whereas no significant effect against the MCF10A cell line. We showed for the first time that hesperidin is a flexible and strong ligand for the MCL-1 receptor. Also, it has the in vitro ability to kill the MDA-MB-231 cell lines without having a significant effect on the MCF10A cell lines. Therefore, hesperidin could be used as a food ingredient to generate functional foods.

## Introduction

Breast cancer is the top cancer in women and the second main cause of cancer death after lung cancer [1]. In early 2020, the World Health Organization (WHO) has declared that the outbreak of breast cancer in the developing world is increasing due to increasing life expectancy, increased urbanization, and adoption of western lifestyles so that it is estimated that 627000 women died from breast cancer that is approximately 15% of all cancer deaths among women. Therefore, finding a way to treat this deadly disease is very important.

Molecular docking techniques aim to predict the best matching binding mode of a ligand to a macromolecular partner, and Molecular dynamics (MD) is a computational technique that simulates the dynamic behavior of molecular systems as a function of time [2]. Hence, performing such techniques needs a macromolecule as a receptor and a ligand. In the breast cancer cells, the estrogen receptors are the main cause of this disease and are expressed in 75% of them [3]. Among these receptors, the estrogen receptor alpha (ERα) is expressed in a low fraction of normal breast epithelium cells and it is for female reproductive organs [4]. A study has shown that the expression of this receptor significantly increases in breast cancer up to 80% of cells [5]. Also, the human epidermal growth factor receptor (HER) generates a cascade of responses able to advance the formation and progression of breast cancer [6]. Therefore, the activation of HER receptors leads to modification in the behavior of normal cells through the final signals for cellular proliferation, anti-apoptosis, angiogenesis, and metastasis [6, 7]. On the other hand, the ability of cancer cells to evade apoptosis is a crucial feature for them, so that they frequently dysregulate the intrinsic apoptotic pathway to preserve tumor cell survival through upregulation of anti-apoptotic Bcl-2 family proteins, including Bcl2-A1, Bcl-2, Bcl-xL, Bcl-w, and Mcl-1 [8]. Therefore, these protein targets can be used as therapeutics target to treat breast cancer. Today, several market drugs such as tamoxifen, raloxifene, toremifene, and fulvestrant for the treatment of breast cancer are available, but each has its limitations, which cause irreversible side effects [9].

The use of natural products in drug discovery and food industries possess several advantages, including unmatched chemical diversity with structural complexity and biological potency; occupy a complementary region of chemical space; the generation of libraries of natural product analogs, which might have enhanced drug-like properties; optimizing the regulation of natural product biosynthesis; lead to the discovery and better understanding of targets and pathways involved in the disease process, and can go straight from hit to drug [10]. Olivero-Acosta et al. [6] were obtained 800 natural compound structures from the NatProd Collection database. They were performed a docking analysis using AtuoDock Vina software to identify interactions between these compounds and human epidermal growth factor receptors. They showed that four natural products named hecogenin acetate, hesperidin, podototarin, and theaflavin are promissory HER receptor inhibitors. According to the above descriptions, we have analyzed these natural products on the estrogen receptor alpha and anti-apoptotic Bcl-2 family proteins using molecular docking and molecular dynamics simulation methods to identify the interactions and stability between them. Therefore, this study aimed to discover a more selective compound targeting breast cancer and establish a nanoliposome encapsulation to increase solubility and biocompatibility of the hesperidin for using as a therapeutic agent.

## Materials and methods

### *In silico* experiments

#### Protein preparation

The 3D structures of protein targets named BCL-2 (PDB ID: 4MAN), BCL-W (PDB ID: 2Y6W), MCL-1 (PDB ID: 5FDO), and ERα (PDB ID: 1G50) were retrieved from the RCSB Protein Data Bank. These files were introduced to AutoDock Tools 1.5.7 (the Scripps Research Institute, La Jolla, CA, USA) software to remove water molecules, and add polar hydrogen atoms, Kollman partial charges, and AD4 type atoms. Finally, the protein files were written as.pdbqt file format for docking analysis.

#### Ligand preparation

The 3D structures of four natural products named hecogenin acetate (CID: 101906), hesperidin (CID: 10621), podototarin (CID: 5320650), and theaflavin (CID: 135403798) were retrieved from the chemical database of PubChem. The downloaded files were converted to.pdb file format using the PyMOL software (Molecular Graphics System, Version 2.0 Schrödinger, LLC.). The ligand files were prepared using AutoDock Tools 1.5.7 (the Scripps Research Institute, La Jolla, CA, USA) software and finally written as.pdbqt file format for docking analysis.

#### Molecular docking analysis

Molecular docking analysis was performed by using AutoDock Vina 1.1.2 (the Scripps Research Institute, La Jolla, CA, USA) software [11] in five independent runs for each ligand. The ligands were docked into the target structures using AutoDock Vina 1.1.2 with grid box values shown in Table 1. The results were evaluated to identify the lowest binding energy and calculate the inhibition constant of the interactions.

**Table 1:** The grid box values used for molecular docking analysis.

Protein target	Size points (x × y × z)	Spacing center (Å)
BCL-2	80 × 80 × 50	0.375
BCL-W	100 × 90 × 100	0.375
MCL-1	120 × 126 × 120	0.647
ERα	120 × 120 × 120	0.375

#### Protein-ligand complex visualization

The protein-ligand complexes were visualized using the Discovery Studio Visualizer 20.1 software. Using this software, the polar and hydrophobic interactions between ligand and target were characterized, and 2D and 3D illustrations of such interactions were generated.

#### Molecular dynamics simulation

Molecular dynamics simulation of hesperidin-ERα and hesperidin-Mcl-1 complexes were performed using GROMACS version 2018 software. The CGenFF server and the CHARMM36 force field was used to generate the topology files for ligand and protein, respectively [12]. Each complex was solvated in a 1.0 nm triclinic box using the TIP3P water model and neutralized using NA and CL ions. Also, each complex was simulated at 310.15 K and a pressure of 1 bar. Afterward, the equilibrated complexes were set up to produce molecular dynamics simulation for 200 ns. The resulting trajectories were analyzed to prove the stability and compactness of the structure by generating the root mean square deviation (RMSD), root mean square fluctuation (RMSF), the radius of gyration (Rg), and solvent accessible surface area (SASA) using the GROMACS program.

### *In vitro* experiments

#### Hesperidin extraction

Hesperidin was extracted from the albedo of Persian sweet lemon (*Citrus limetta*), according to the previously described method [13], with some modifications. Briefly, the dry albedo was powdered in liquid nitrogen and then mixed with methanol in the ratio of 1:5 (w/v). The homogenous was placed in a water bath at 55°C for 3 h with shaking. The supernatant was collected and 60 mL of methanol was again added to residues. After heating at 55°C for 30 min, two supernatants were combined and filtered. The solvent was evaporated and the mixture of dichloromethane: water in the ratio of 1:1 was added to extract. This step was repeated and finally, hesperidin crystals were collected by a paper filter.

#### RP-HPLC analysis of extracted hesperidin

The extracted hesperidin was analyzed using an analytical reverse-phase HPLC equipped with a C18 column (250 mm × 4.6 mm, 5 μm, 100 Å; Knauer Azura Chromatography, Berlin, Germany) to determine its purity. The separation was performed at a flow rate of 0.25 mL/min and a column temperature of 40°C. The column was eluted with a gradient of 0–60% mobile phase B (MeOH) for 20 min, 60–90% mobile phase B for 6 min, and 100% mobile phase B for 10 min. The mobile phase A was water and 0.1% formic acid. The elution was monitored at 285 nm using a UV detector.

#### Preparing hesperidin-loaded nanoliposomes

The hesperidin-loaded nanoliposomes were prepared according to the previously described method [14], with slight modifications. Briefly, 2% soy lecithin solution was prepared and agitated for 12 h on the stirrer. 10 mg of hesperidin was added to the mixture and vortexed for 15 min. Subsequently, the mixture was sonicated at 80% of full power for 5 min (1 s on, 1 s off) using the probe sonicator (SONOPLUS HD-4200, Bandelin, Germany). The nanoliposome suspension was filtered with a polycarbonate filter (0.22 μm) and stored at 37°C in dark for more analysis. The mixture without hesperidin was used as control.

#### Physicochemical properties of nanoliposome

The particle size and zeta potential of nanoliposome were evaluated by dynamic scattering light using an SZ-100 nanopartica series instrument (Horiba, Japan). The suspension was diluted in ddH_2_O (1:40) and placed in a vertical cylindrical cell. The measurement was carried out at a scattering angle of 173° relative to the source and 25°C with a refractive index of 1.34 [15].

#### Entrapped efficiency of hesperidin

The nanoliposome suspension was centrifuged at 30000 g for 1 h. The supernatant was collected and then the amount of free hesperidin (unencapsulated) was measured using the AlCl_3_ method according to the previously described method [16], whereas the various concentrations of quercetin were used as a standard curve. The entrapped efficiency (EE) was calculated using the following equation: EE% = Ci–Cs / Ci × 100, where Ci and Cs were the initial amount of hesperidin and the unencapsulated amount of hesperidin in the supernatant, respectively.

#### TEM analysis of nanoliposome

The concentration of nanoliposome in suspension was reduced through 10-folds dilution with ddH_2_O. To negative staining, an equal volume of diluted suspension was mixed with 2% ammonium molybdate solution and kept for 3 min at 25°C. Subsequently, a drop of the stained suspension was placed on Formvar-carbon coated copper grid for 5 min and then the excess liquid was drawn off using filter paper. After air-drying at 25°C, the morphology of nanoliposomes was evaluated by TEM (LEO 906 E, Philips, Germany) operating at 200 kV [15].

#### Cytotoxicity assay

Cytotoxicity assay of hesperidin was evaluated against the MDA-MB-231 human breast cancer cell line and the MCF10A normal cell line using MTT (3- [4, 5-dimethylthiazol-2-yl]-2, 5 diphenyl tetrazolium bromide) assay method, with some modification [17]. The cancerous and normal cell lines were respectively cultured in a fresh RPMI-1640 and DMEM medium containing 10% fetal bovine serum, 100 U/mL penicillin, and 100 μg/mL streptomycin at 37°C in a humidified incubator with 5% CO_2_. After sequential passages, 200 μL of medium containing 1 × 10^4^ cells was poured into each well of the 96-well plate and incubated in the above conditions for 24 h. Afterward, the various concentrations of encapsulated nanoliposomes or hesperidin (10, 20, 40, 60, and 80 μg/mL) were replaced with medium and incubated in the above conditions for 24 h. The treatments were removed, and the cells were washed with PBS buffer (pH 7.2). 100 μL of medium containing 0.5 mg/mL MTT reagent was added into each well and incubated in the above conditions for 4 h. Finally, 100 μL of DMSO was replaced with MTT solution and incubated in the above conditions for 15 min with shaking. The absorbance was read using a microplate reader (Bio-Rad, Richmond, CA, USA) at 570 nm. Unencapsulated nanoliposome and fresh medium were used as a negative control. Triton X-100 was used as a positive control. The IC_50_, GI_50_, and LC_50_ values were calculated according to the previously described method [18].

### Statistical analysis

All *in vitro* experiments were performed in triple repeats and analyzed using SAS9.4 software. A mean comparison analysis was performed by Tukey test. Plotting the graphs and calculating the IC_50_, GI_50_, and LC_50_ values were done with the GraphPad Prism software.

## Results and discussion

### Molecular docking

The Gibbs free energy (kcal/mol) is suggesting an equilibrium state and complex stability in the protein-ligand binding process [19]. In this study, the results of molecular docking showed that the natural product of hesperidin tends to bind to BCL-W, MCL-1, and ERα target proteins with the lowest binding energy of -9.2, -10.1, and -9.5 kcal/mol, respectively (Table 2). Also, the natural product of theaflavin was bound to the BCL-2 target protein with the lowest binding energy of -8.3 kcal/mol (Table 2). In addition, the inhibition constant of each ligand was calculated from the binding energy according to the following equation: ΔG = RT*ln*Ki, whereas ΔG, R, T and Ki are related to the binding energy (cal/mol), gas constant (1.987 cal/K mol), temperature (310.15 K), and inhibition constant (Table 2). Consequently, the hesperidin-MCL-1 and hesperidin-ERα complexes were selected for the stability analysis using molecular dynamics simulations.

**Table 2:** The binding potency and inhibition constant of desired ligands with the target proteins.

Target protein	Ligand	Binding energy (kcal/mol)	Inhibition constant (μM)
BCL-2	Hecogenin acetate	- 7.8	1.91
	Hesperidin	- 8.0	1.37
	Podototarin	- 7.5	3.18
	Theaflavin	- 8.3	0.823
BCL-W	Hecogenin acetate	- 7.6	2.68
	Hesperidin	- 9.2	0.18
	Podototarin	- 8.1	1.16
	Theaflavin	- 8.5	0.586
MCL-1	Hecogenin acetate	- 8.6	0.494
	Hesperidin	- 10.1	0.039
	Podototarin	- 8.4	0.694
	Theaflavin	- 8.7	0.421
ERα	Hecogenin acetate	- 9.2	0.18
	Hesperidin	- 9.5	0.108
	Podototarin	- 7.8	1.91
	Theaflavin	- 9.0	0.253

**Fig 1:**
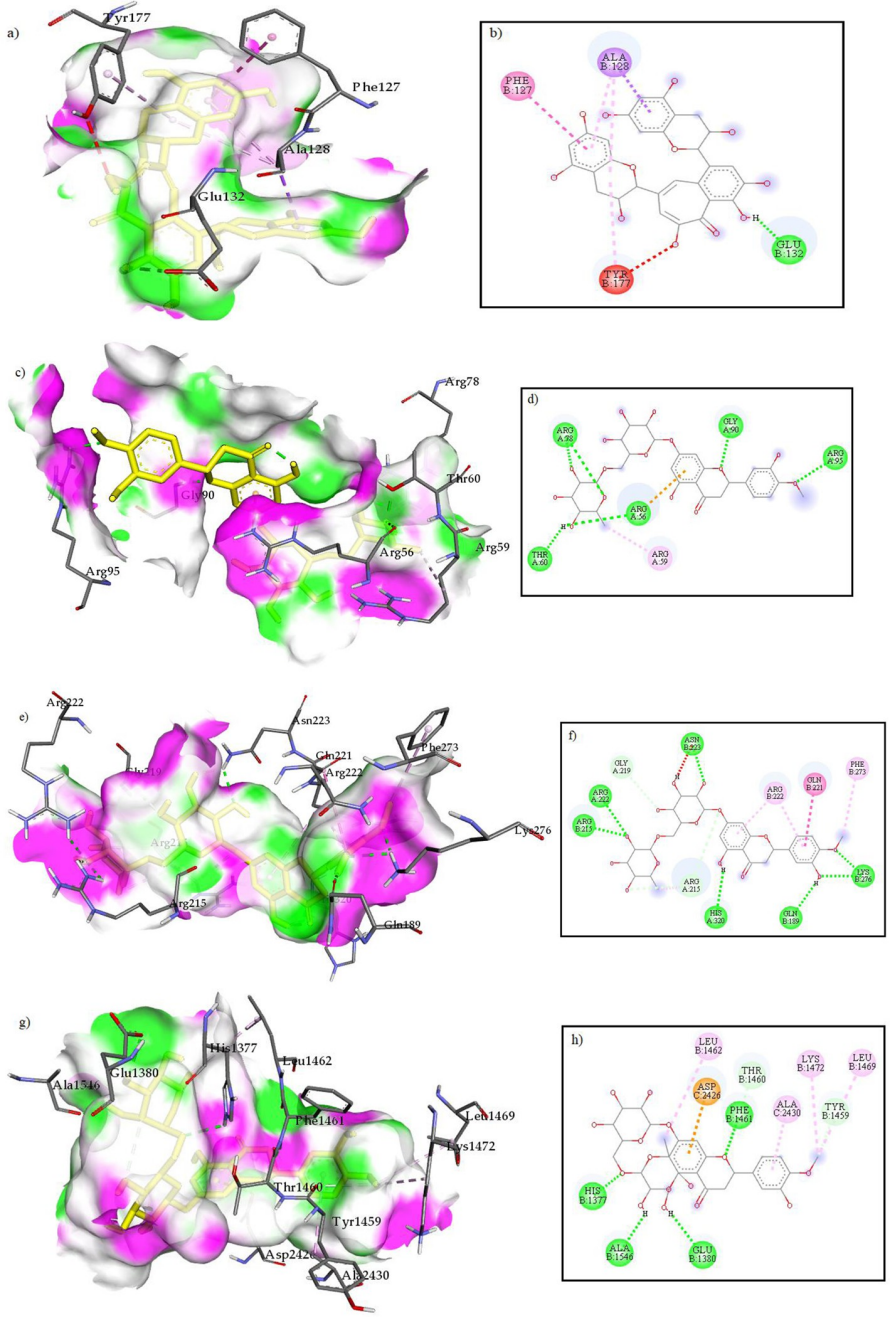
The 3D and 2D illustrations of the best binding mode and interacting residues of theaflavin-BCL-2 (a, b), hesperidin-BCL-W (c, d), hesperidin-MCL-1 (e, f), and hesperidin-ERα (g, h) complexes.

The best binding mode and interacting residues for four complexes of theaflavin-BCL-2, hesperidin-BCL-W, hesperidin-MCL-1, and hesperidin-ERα were shown in Fig 1. As previously has shown, the active site of BCL-2 protein consists of Arg10, Val13, Met14, Trp28, Ala30, Gly31, Leu94, Ala97, Gly98, Asp100, Phe101, Tyr105, Asp108, Phe109, Met112, Val130, Leu134, Trp141, Gly142, Arg143, Ile144, Val145, Ala146, Phe147, Glu149, Phe150, Val153, Asp168, Ala171, Leu172, Thr175, Phe195, and Tyr199 residues [20–22]. The theaflavin in the binding site of BCL-2 interacted through one hydrogen interaction with Glu132, one Pi-Alkyl interaction with Ala128, one Pi-Pi T-shaped interaction with Phe127, and one Pi-Sigma interaction with Ala128 (Fig 1A and 1B). The hesperidin interacted with BCL-W, MCL-1, and ERα target proteins through 8, 16, and 11 interactions, respectively. In the case of the binding site of BCL-W protein consisting of Asp153, Arg160, Val162, Trp167, Val170, Arg171, Arg177, Ala179, and Leu180 residues [23], the interactions in the hesperidin-BCL-W complex were through two hydrogen interactions with Arg78, one hydrogen interaction with each of Arg56, Thr60, Gly90, and Arg95, one Pi-Cation interaction with Arg56, and one Alkyl interaction with Arg59 (Fig 1C and 1D). The binding site residues of MCL-1 protein were determined as His224, Ala227, Phe228, Met231, Leu235, Ile237, Leu246, Val249, Met250, Val253, Phe254, Asp256, Asn260, Arg263, Thr266, Leu267, Phe270, Gly271, Val274, Ile294, and Leu298 [24, 25]. According to this, the interacting residues in the hesperidin-MCL-1 complex were Gln189, Arg215, Gly219, Gln221, Arg222, Asn223, Phe273, Lys276, and His320, so that these interactions included seven hydrogen interactions, three Carbon hydrogen interactions, one Amide-Pi Stacked interaction, and three Alkyl and Pi-Alkyl interactions (Fig 1E and 1F). Also, the interacting residues of hesperidin on ERα active site, consisting of residues Leu349, Ala350, Leu384, Leu387, Leu391, Arg394, Phe404, Ile424, Gly521, His524, Leu525, and Met528 [4, 26], were included His1377, Glu1380, Phe1461, and Ala1546 through hydrogen interactions; Leu1462, Leu1469, Lys1472, and Ala2430 through Alkyl and Pi-Alkyl interactions; Tyr1459 and Thr1460 through Carbon hydrogen interaction; and Asp2426 through Pi-Anion interaction (Fig 1G and 1H). Although ligands have not exactly interacted with binding site residues of receptors, their binding patterns indicate that they may act as partial agonists or allosteric modules, which require more investigation using the pharmacological assay. Generally, a lower binding energy shows more rational and stable interaction between ligand and receptor. However, hesperidin-MCL-1 complex was the most stable complex with the best binding affinity of -10.1 kcal/mol.

The various studies have proved that hesperidin has a capacity to induce cancer cell death including breast, lung, colon, liver, and gastric cancers [6, 19]. Also, a study showed that the hesperidin significantly inhibits the colony formation of MCF7 cells [27]. On the other hand, it has been reported that the apoptotic effects of hesperidin are associated with altered ratios of pro-/antiapoptotic proteins, caspase activation, c-Jun N-terminal kinase (JNK) pathway activation, and caspase-independent pathways [28]. Therefore, according to the results of previous studies and molecular docking in this study, the selection of hesperidin as a potential inhibitor of breast cancer to evaluate its stability using molecular dynamics simulations and perform the *in vitro* studies can be useful to help to produce an anticancer drug after *in vivo* and clinical experiments.

### Molecular dynamics simulations

**Fig 2:**
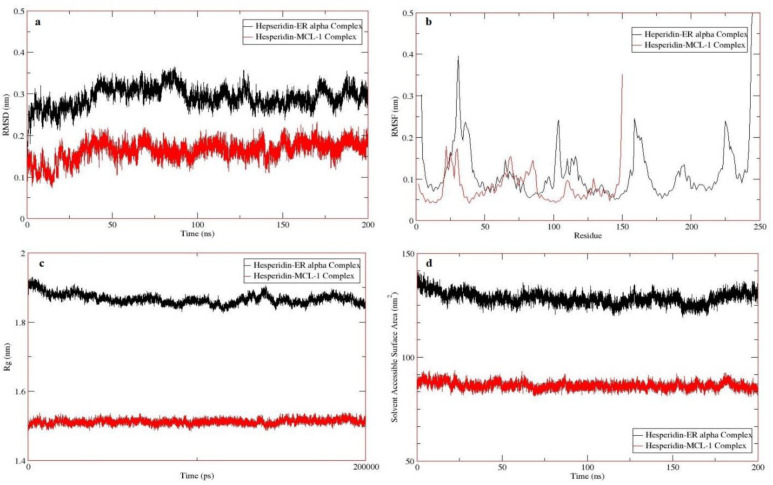
Molecular dynamics simulation of the hesperidin-MCL-1 and hesperidin-ERα complexes at 310.15 K for 200 ns. a) RMSD plot, b) RMSF plot, c) Rg plot, and d) SASA plot.

Molecular dynamics (MD) is a computer simulation method for analyzing the physical movements of atoms and molecules. According to the definition, the stability of hesperidin-MCL-1 and hesperidin-ERα complexes were evaluated using the Gromacs software at 310.15 K for 200 ns. RMSD calculates the average of the whole particle for every moment [29], therefore structural changes and deviations in both complexes are shown in Fig 2. The average RMSD value of hesperidin-MCL-1 and hesperidin-ERα complexes is 0.16 ± 0.023 and 0.29 ± 0.025 nm, respectively. The lower RMSD indicates more stability of the complex [30]. The results showed that the stability of hesperidin in the hesperidin-MCL-1 complex is higher than the hesperidin-ERα complex. According to the RMSD plot (Fig 2A), although the hesperidin-ERα complex was shown slight fluctuations at 125–160 ns, it can be concluded that this complex was partly stabilized. On the other hand, the hesperidin-MCL-1 complex has completely stabilized at 0.16 nm (Fig 2A).

The flexibility of complexes was checked by the RMSF plot (Fig 2B). The average RMSF is made on the total time per residue [29], which calculated 0.08 ± 0.038 and 0.12 ± 0.092 nm for hesperidin-MCL-1 and hesperidin-ERα complexes, respectively. The most flexible parts of the protein structure are loop structures, and by nature, they are the most fluctuated regions in protein complexes [22]. On the other hand, more RMSF indicates a more flexible complex. Therefore, the hesperidin-MCL-1 complex is more stable.

The radius of gyration (Rg) determines the compactness of protein and this factor is different in each of the classes of protein [31]. The average Rg value was 1.51 ± 0.007 and 1.86 ± 0.014 nm for hesperidin-MCL-1 and hesperidin-ERα complexes, respectively (Fig 2C). It indicates that the protein folding is lower than the initial time in the hesperidin-ERα complex and has slightly increased at the end of the simulation, but it has remained almost constant during the simulation for the hesperidin-MCL-1 complex. On the other hand, the SASA analysis is closely related to Rg analysis [22]. However, the average SASA value was 86.42 ± 1.74 and 128.43 ± 2.95 nm^2^ (Fig 2D). Since the SASA plot shows the connection of protein with the solvent environment and it depends on protein size, it can be concluded that the higher the level of solvent access, the lower the protein folding, resulting in more amino acids available to interact with the ligand.

### Purity evaluation of extracted hesperidin

**Fig 3:**
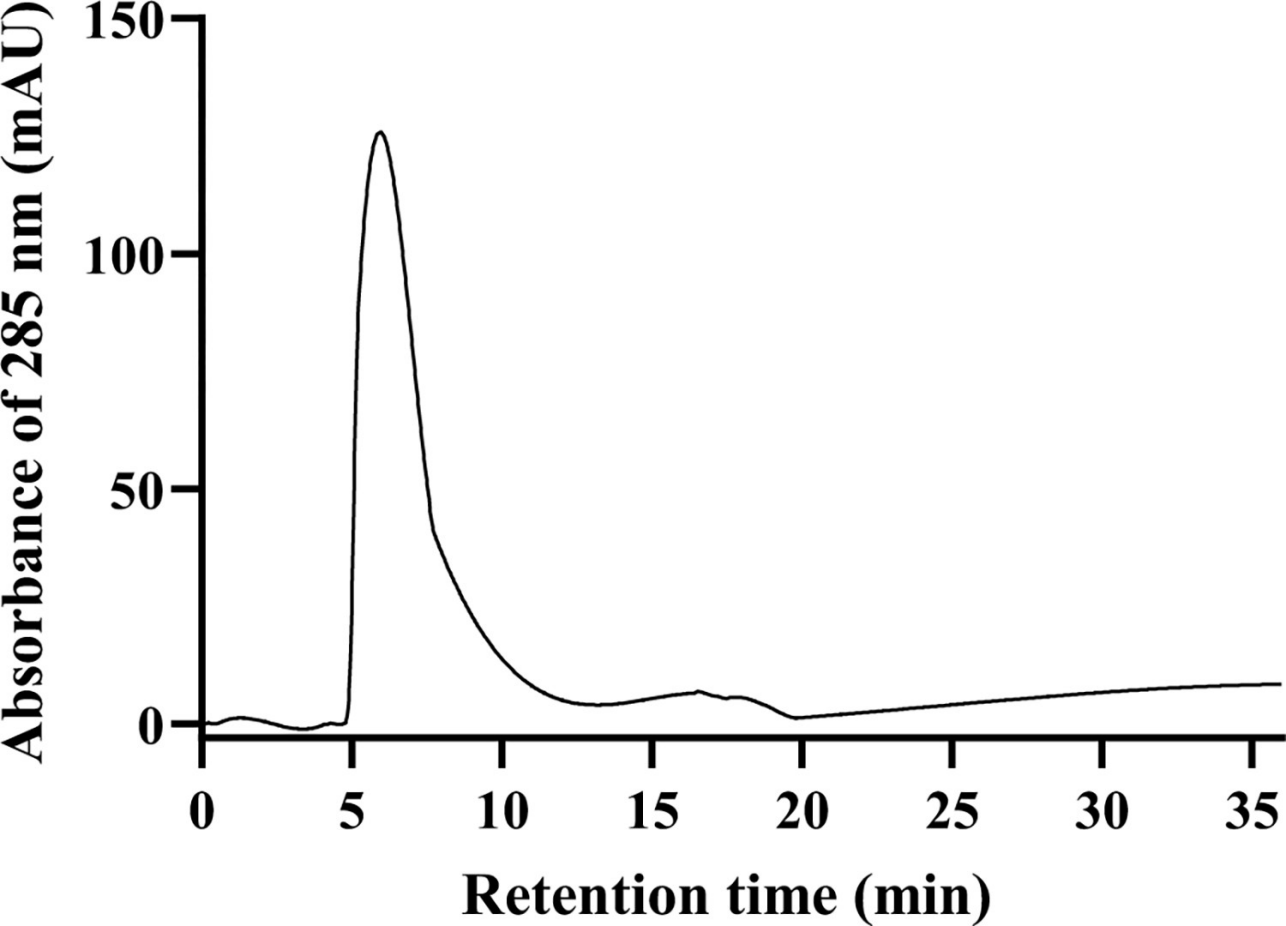
The RP-HPLC profile of hesperidin isolated from C. limetta with yielded of 98.8%.

The RP-HPLC chromatogram was shown a sharp peak at the retention time of 5.95 min (Fig 3). In a similar extraction method, Victor et al. [13] reported that the purity of extracted hesperidin with the yield of 2.5% has been 89.4% using RP-HPLC, whereas the extracted hesperidin in this study had the purity of 98.8% with the yield of 1.72%.

### Physicochemical properties of nanoliposomes

The particle size of nanoliposomes is an essential quality control assay and a prominent factor for determining entrapment efficiency, *in vivo* nanoliposome applications, and the release of bioactive compounds [15, 32]. The particle size of nanoliposomes was measured using dynamic light scattering after sonication. The z-average of nanoliposomes before and after encapsulation with hesperidin was 137.5 ± 1.01 and 123.03 ± 0.95 nm, respectively (Table 3). The decrease in nanoliposomes size after encapsulation indicates a strong interaction between hesperidin and lecithin, which results in a compaction of the core of the nanoliposomes. This interaction can be between the acyl chains of the lecithin bilayer and the hesperidin molecule. On the other hand, Span is an index for measuring particle size distribution in solutions and determine from the following equation: (d_90_ –d_10_)/ d_50_, so that d_90_, d_10_, and d_50_ are related to 90, 10, and 50% intensity on a relative cumulative particle size distribution curve. The Span index was 0.74 ± 0.08 and 0.55 ± 0.07 for nanoliposomes before and after encapsulation, respectively. It shows that the samples are of narrow distribution. In addition to physical parameters, the size of nanoliposomes depended on fatty acid composition, lipid classes, and the surface-active properties of lecithin [14].

**Table 3:** The particle size and zeta potential of nanoliposomes before and after encapsulation.

Nanoliposome	Particle size (nm)	Span	Zeta potential (mV)
Before encapsulation	137.5 ± 1.01^a^	0.74 ± 0.08^a^	- 50.33 ± 1.26^b^
After encapsulation	123.03 ± 0.95^b^	0.55 ± 0.07^b^	- 63.53 ± 1.35^a^

Note: a and b letters show a significant level at a *P-value* of ≤ 0.01.

According to Table 3, the surface charge of nanoliposomes was increased after hesperidin-encapsulation. The zeta potential lower than– 30 and higher than 30 mV indicates the stability of systems. Therefore, hesperidin loaded and unencapsulated nanoliposomes possess high stability. The increase of zeta potential after hesperidin-encapsulated may be due to physicochemical properties of hesperidin such as *log P* (- 0.31). On the other hand, the negative charge of soy lecithin nanoliposomes is due to the presence of negatively charged phospholipids including phosphatidylserine, phosphatidic acid, phosphatidylglycerol, phosphatidylinositol in its lipid composition, which all of them have a negative charge in physiological pH [14, 33].

### Entrapped efficiency

The bioavailability and solubility of bioactive compounds such as hesperidin can enhance using nanoliposome formulations [32, 34]. The entrapped efficiency of hesperidin in soy lecithin nanoliposomes was calculated at 92.02 ± 1.08%. It shows that the solubility of hesperidin loaded on nanoliposomes was increased so that the solubility of hesperidin in water is 4.93 μg/mL [35]. Therefore, the high entrapped efficiency of hesperidin might be because of observing the significant differences of charge, size, and zeta potential before and after encapsulation.

### TEM analysis

**Fig 4:**
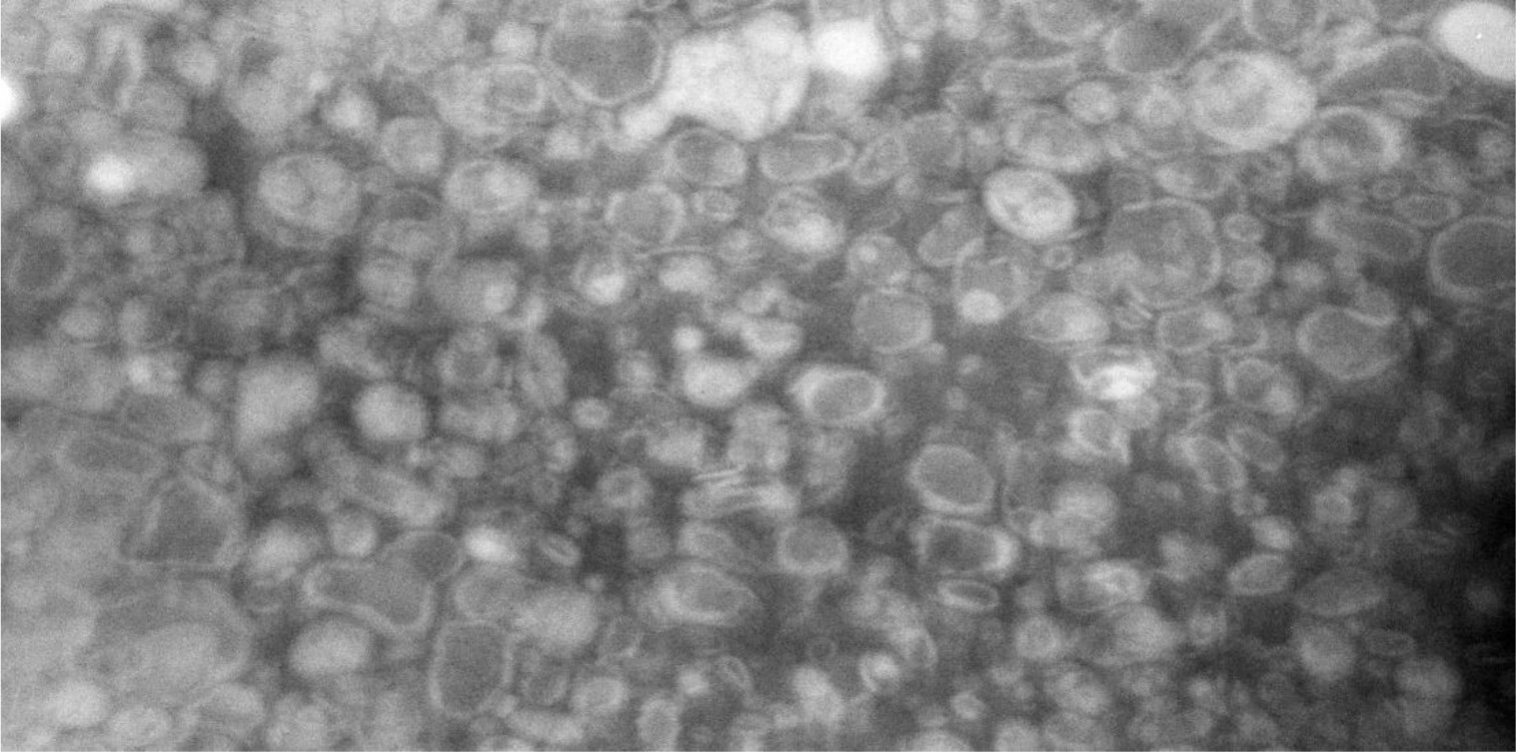
The transmission electron microscopy (TEM) image of nanoliposomes composed of soy lecithin.

The morphology of nanoliposomes was evaluated by TEM analysis. The results showed a nanometric round shape and unilamellar vesicles, which are suitable for creating drug delivery systems. In addition, the bilayer structures are clearly visible in the TEM image (Fig 4), which confirms the formation of liposomes.

### Cytotoxicity assay

**Fig 5:**
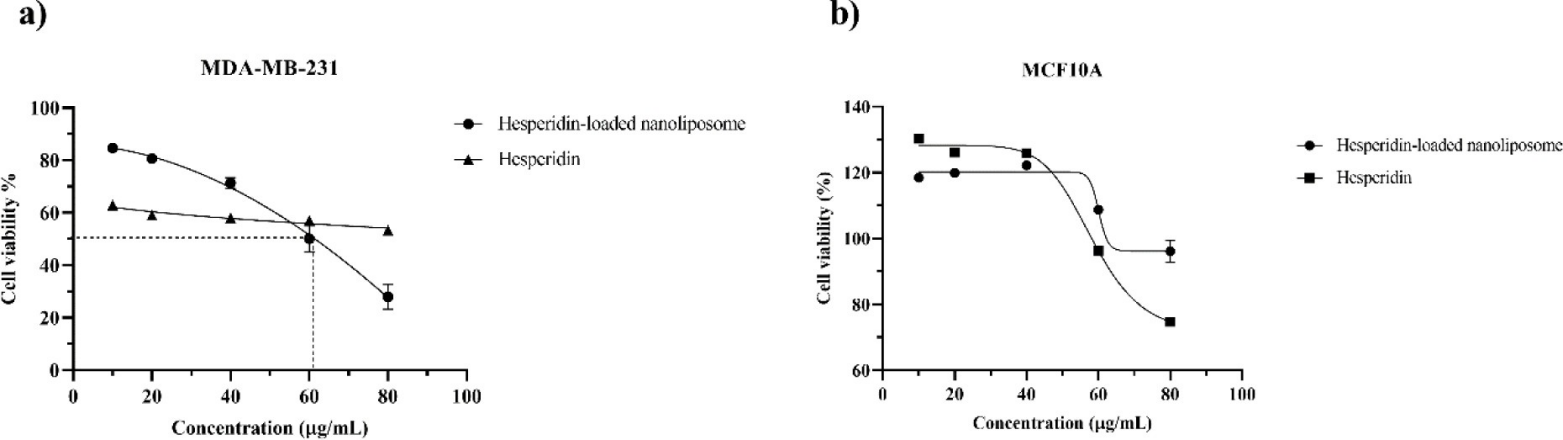
The cytotoxicity assay of hesperidin-loaded nanoliposomes and hesperidin against the MDA-MB-231 (a) and the MCF10A (b) cell lines using the MTT assay method. Dotted lines on the graph show the IC_50_ value.

The cytotoxicity assay showed that hesperidin-loaded nanoliposomes were able to kill the MDA-MB-231 cell line. According to Fig 5, the cell viability percentage decreases with increasing concentration. In contrast, the hesperidin had a killing ability higher than the hesperidin-loaded nanoliposomes at concentrations from 10 to 60 μg/mL. This ability was more for hesperidin-loaded nanoliposomes at a concentration of 80 μg/mL. It shows that the nanoliposomes may have been able to release more hesperidin at higher concentrations. The IC_50_ value of hesperidin-loaded nanoliposomes and hesperidin was calculated 62.93 and ˃ 80 μg/mL (Fig 5), while unencapsulated nanoliposomes were shown no cytotoxicity effect (Data no shown). Therefore, the nanoliposome formulation has increased the effectiveness of hesperidin in killing cancerous cells. Also, the GI_50_ and LC_50_ values of hesperidin-loaded nanoliposome and hesperidin were calculated ˃ 80 μg/mL in both (Data no shown). In contrast, hesperidin-loaded nanoliposomes and hesperidin showed no cytotoxicity effect against MCF10A, although hesperidin alone was inhibited the growth of normal cells at a concentration of 80 μg/mL with a cell viability percentage of 74.73% (Fig 5). Generally, the IC_50_, GI_50_, and LC_50_ values of hesperidin-loaded nanoliposome and hesperidin against the MCF10A cell line were calculated ˃ 80 μg/mL for all. On the other hand, nanoliposomes have protected the MCF10A cell line against the cytotoxic effects of hesperidin (Fig 5).

The previous studies have shown that hesperidin interacts with numerous recognized cellular targets and inhibits cancer cell proliferation by inducing apoptosis and cell cycle arrest [36]. However, the cytotoxicity effects of hesperidin were proved against breast cancer [37]. Since hesperidin is water-insoluble, therefore it is necessary to find a way to increase its solubility, bioavailability, and in turn the cytotoxicity effects. One of these ways is nano-formulations. In a study, the cytotoxicity effect of hesperidin against the MDA-MB-231 cell line was increased after loading on gold nanoparticles with an IC_50_ value of 70–75 μg/mL, so that IC_50_ value of hesperidin before nanoencapsulation was calculated ˃ 125 μg/mL [38]. In addition, hesperidin loaded on nanoparticles showed no significant cytotoxicity effect (IC_50_ value of ˃ 125 μg/mL). In another study, the cytotoxicity of hesperidin was evaluated against the MCF-7 cell lines and the IC_50_ value was calculated 100–140 μg/mL [39]. Also, hesperidin-loaded PLGA nanoparticles and hesperidin-loaded nano-emulsions have improved the cytotoxicity effect of hesperidin [40, 41]. However, using the nanoliposome composed of soy lecithin for loading hesperidin has not been investigated. Since lecithin acts as a type of membrane phospholipid, it maintains membrane fluidity and facilitates drug absorption [42]. On the other hand, Therapeutic efficacy of drugs with poor oral absorption is improved through lecithin-mediated formulations [43]. Therefore, according to previous studies and our results, nanoencapsulation of hesperidin could be a way for increasing the bioavailability and enhancing the cellular effect on cell proliferation, especially the MDA-MB-231 cell line.

## Conclusion

The use of natural products can be useful for the therapeutic and nutritional purposes of humans. Numerous studies have shown that natural products are a good alternative to synthetic and chemical drugs because they have no side effects and are of natural origin. Based on the results of the present study, we can also remark that natural compounds such as hesperidin have the ability to become a unique and natural drug. Therefore, in order to achieve this promising goal, extensive *in vivo* and clinical studies are needed, although some researches have been started in this field. In this study, we showed that hesperidin has a strong binding power to MCL-1 receptors and also the stability of this binding is high and flexible. In addition, *in vitro* studies have shown that it has the ability to kill the MDA-MB-231 breast cancer cell lines without having a significant effect on the MCF10A normal cell lines. Therefore, hesperidin can be a promising target for the treatment of breast cancer as a drug or food additive.

## Supporting information

S1 Data (XLSX)
